# Perinatal Outcomes of Multiple Births in Southwest Nigeria

**Published:** 2011-12

**Authors:** Bolajoko O. Olusanya

**Affiliations:** ^1^Institute of Child Health and Great Ormond Street Hospital for Children NHS Trust, University College London, 30 Guilford Street, London, UK; ^2^Maternal and Child Health Unit, Department of Community Health and Primary Care, College of Medicine, University of Lagos, Surulere, Lagos, Nigeria

**Keywords:** Cross-sectional studies, Multiple gestations, Perinatal outcomes, Retrospective studies, Twins, Nigeria

## Abstract

Compared to singletons, multiple births are associated with a substantially-higher risk of maternal and perinatal mortality worldwide. However, little evidence exists on the perinatal profile and risk of neurodevelopmental disabilities among the survivors, especially in developing countries. This cross-sectional study, therefore, set out to determine the adverse perinatal outcomes that are potential markers for neurodevelopmental disabilities in infants with multiple gestations in a developing country. In total, 4,573 mothers, and their 4,718 surviving offspring in an inner-city maternity hospital in Lagos, Nigeria, from May 2005 to December 2007, were recruited. Comparisons of maternal and infant outcomes between single and multiple births were performed using multivariable logistic regression and generalized estimation equation analyses. Odds ratio (OR) and the corresponding 95% confidence interval (CI) for each marker were estimated. Of the 4,573 deliveries, there were 4,416 (96.6%) singletons and 157 (3.4%) multiples, comprising 296 twins and six triplets together (6.4% of all live 4,718 infants). After adjusting for maternal age, ethnicity, occupation, parity, and antenatal care, multiple gestations were associated with increased risks of hypertensive disorders and caesarean delivery. Similarly, after adjusting for potential maternal confounders, multiple births were associated with low five-minute Apgar score (OR: 1.47, 95% CI 1.13-1.93), neonatal sepsis (OR: 2.16, 95% CI 1.28-3.65), severe hyperbilirubinaemia (OR: 1.60, 95% CI 1.00-2.56), and admission to a special-care baby unit (OR: 1.56, 95% CI 1.12-2.17) underpinned by preterm delivery before 34 weeks (OR: 1.91, 95% CI 1.14-3.19), birthweight of less than 2,500 g (OR: 6.45, 95% CI 4.80-8.66), and intrauterine growth restriction (OR: 9.04, 95% CI 6.62-12.34). Overall, the results suggest that, in resource-poor settings, infants of multiple gestations are associated with a significantly-elevated risk of adverse perinatal outcomes. Since these perinatal outcomes are related to the increased risk of later neurodevelopmental disabilities, multiple-birth infants merit close developmental surveillance for timely intervention.

## INTRODUCTION

There is extensive evidence that twins and higher-order multiple births worldwide are associated with a substantially-higher risk of maternal and perinatal mortality and morbidity compared to singletons (1-4). Globally, the highest burden of multiple births has been found in sub-Saharan Africa, with an average twining rate of 20 per 1,000 deliveries compared to 10 per 1,000 deliveries in Europe or around 5-6 per 1,000 deliveries in Asia (5-7). Nigeria has the highest prevalence of multiple births worldwide (5,7,8). Twining is a multifactorial phenomenon principally attributable to genetic and environmental factors, such as advanced maternal age and increased parity (3,5,7).

While multiple births are often disproportionately represented among preterm, low birthweight or intrauterine growth-restricted infants, maternal and infant morbidities following multiple pregnancies, unlike perinatal mortality rates, have not been thoroughly described in developing countries (9-13). Additionally, since preterm births, low birthweight, and intrauterine growth restriction are prominent risk factors for adverse neurodevelopmental outcomes worldwide, a better characterization of the perinatal consequences of multiple pregnancies should be helpful in facilitating resource allocation and closer developmental surveillance for infants at risk of developmental delays (6,14-18).

This study was, therefore, conducted to determine the perinatal outcomes of surviving multiples compared to singletons in southwest Nigeria based on the hypothesis that multiple births are at a higher risk of preterm delivery, low birthweight, and intrauterine growth restriction.

## MATERIALS AND METHODS

### Study settings and subjects

This cross-sectional study was conducted at the Island Maternity Hospital (IMH) in Lagos, southwest Nigeria, from May 2005 to December 2007. Nigeria is made up of three major ethnic groups: Hausa in the north, Igbo in the southeast, and Yoruba in the southwest. It is estimated that twins constitute about 5% of all Yoruba births, with reported birth rates of 45-50 twins per 1,000 livebirths and are predominantly dizygotic from spontaneous pregnancies (8). The IMH is an inner-city government hospital providing specialist services to several private and public hospitals within and outside its catchment area. At the time of the study, the hospital had 180 beds for maternity services and a 15-bed special-care baby unit (SCBU) equipped with incubators, stand-alone resuscitation units, suction machines, oxygen concentrators, infusion sets for intravenous fluids (not parenteral nutrition), and phototherapy units. Although the hospital provided tertiary-level maternity services as an accredited institution for residency training in anaesthesia, obstetrics, and gynaecology, it only offered secondary-level neonatal services.

Participants were drawn from a cohort of newborns prospectively recruited under a previously-reported universal newborn hearing screening (UNHS) programme at the hospital and for which ethical approval was obtained from the Lagos State Health Management Board, Nigeria and University College London, UK (19). All newborns who died before or during enrollment into the study were excluded. Informed written consent was obtained from mothers before enrollment in writing or by thumb printing. Non-clinical information was obtained predominantly from the mothers at enrollment while clinical data were obtained from the hospital-records.

### Study variables

The case definition for ‘multiple gestations or births’ was twins or triplets as higher-order births were not recorded during the study period. Independent variables of interest included maternal factors grouped into: sociodemographic factors, such as maternal age, ethnicity (Yoruba and non-Yoruba), education, and occupation; obstetric history, such as parity (primiparous or multiparous), maternal HIV status, antenatal care, and use of herbal drugs in pregnancy; and adverse pregnancy-related events, such as antepartum haemorrhage, premature rupture of the membranes, hypertensive disorders (inclusive of pre-eclampsia, eclampsia, and pregnancy-induced hypertension), prolonged/obstructed labour, and mode of delivery (caesarean or vaginal). Multiple deliveries were regarded as a single parous event. Thus, a woman whose first delivery or viable pregnancy was multiples was considered primiparous. Infant outcomes consisted of preterm birth (<37 completed weeks of gestation), extreme-to-moderate preterm birth (<34 completed weeks of gestation), low birthweight (<2,500 g), very low birthweight (<1,500 g), intrauterine/foetal growth restriction (IUGR), and ‘perinatal’ comprising birth asphyxia (indexed by Apgar scores of <7 at one minute and five minutes), suspected neonatal sepsis (used collectively for septicaemia, meningitis, and pneumonia), and hyperbilirubinaemia (requiring phototherapy). Gestational age was based on the number of days between the first day of the last menstrual period (LMP) and the date of delivery expressed in completed weeks after the LMP while IUGR was determined by birthweight below 2 standard deviation (SD) of the mean birthweight for each gestational age based on a previously-validated foetal growth curve for this study population (20). Additionally, admission to an SCBU aimed at capturing any other undocumented/undiagnosed morbidities, mode of feeding (exclusive or non-exclusive breastfeeding), and gender were included. Within the context of the substantive UNHS programme, the hearing screening status (pass or fail) of the infant was also considered based on a two-stage hearing screening protocol consisting of a first-stage screening with transient evoked otoacoustic emissions (TEOAE), followed by a second-stage screening with automated auditory brainstem response (AABR) test as previously reported (19).

### Statistical analysis

The SPSS software (version 16.0) (SPSS Inc., Chicago, IL, USA) was used for all statistical analyses. Univariate associations between multiple gestations and maternal/perinatal outcomes were explored with Pearson's χ^2^ or Fisher's exact tests (categorical variables) and with Student's *t*-test (continuous variables). The strength of the association was estimated with odds ratio (OR) and 95% confidence intervals (CIs) as an approximation of the relative risk. The regression modelling approach was guided by the hierarchical inter-relationships among the independent variables as recommended by Victora *et al*. (21). To account for the clustering effect of multiple births, the generalized estimating equation (GEE) method was used for deriving the significant neonatal outcomes after adjusting for all significant maternal factors (22). This method allows data for multiple births to be treated as repeated measures of the same birth. The significant trend in the neonatal outcomes across the three gestational age-groups: extreme-to-moderate preterm (<34 weeks), late-preterm (34-36 weeks), and term (≥37 weeks) was determined by the linear-to-linear χ^2^ for the categorical variables and Kruskal-Wallis test for the continuous variables. The possible mediating effects of prematurity, low birthweight, and IUGR on rates of birth asphyxia, suspected neonatal sepsis, and hyperbilirubinaemia were further tested by enlisting them as covariates in the GEE analyses. Collinearity among explanatory variables was verified by tolerance value and variance inflation factor (1/tolerance).

## RESULTS

In total, 4,573 mothers and their 4,718 surviving offspring (51.8% male) were enlisted over the study period. Of 157 mothers with multiple pregnancies, 155 delivered twins, and two delivered triplets, of which 296 were surviving twins and six were triplets. Thus, 14 infants did not have a surviving second twin. The pattern of sex concordance showedthat 182 twins had same sex, 100 twins and all six triplets had opposite sex while the sex-pairing in the infants without second surviving twin could not be determined. The crude rate for multiple gestations in the study was at least 3.4% (157/4,573), 98.7% of which were twins. The zygosity of multiple gestations was unknown. The sociodemographic and obstetric history of the mothers is presented in Table 1. The study subjects were predominantly (75.7%) of the Yoruba tribe. Mothers with multiple gestations were significantly likely to be aged 25 years or older, engaged in a full-time occupation, multiparous, and without antenatal care. The risk of multiple pregnancies (estimated by ORs) increased with advanced maternal age and higher parity. When antenatal care was stratified by parity, multiparous mothers with multiple pregnancies were found to have over 70% increased risk (OR: 1.70, 95% CI 1.20-2.40, p=0.002) of not attending antenatal clinics compared to primiparous. There were no significant differences in the mean gestational age between mothers with multiple gestations and singletons. The adverse pregnancy-related events are presented in Table 2. Mothers with multiple gestations were significantly more likely to be associated with hypertensive disorders (p<0.001) and caesarean delivery (p=0.002) but less likely to have prolonged and/or obstructed labour (p=0.050) after adjusting for maternal age, ethnicity, occupation, and lack of antenatal care. Although ethnicity did not show any significant association in the univariate analysis, this factor was included in all the multivariable analyses based on the established high prevalence of twining among the Yoruba tribe and the fact that traditional practices relating to pregnancy and childbirth which may affect the outcomes varied across the tribes.

Table 3 shows that multiple births were significantly more likely to be female (p=0.018), with significantly lower mean birthweight of 2.54±0.53 kg compared to 3.20±0.65 kg for singletons (p<0.001). Only 17 (0.4%) infants in the total study population had congenital abnormalities, such as craniofacial defects or other physical deformities at birth, of which three (17.6%) were twins (p=0.091) (data not shown). After adjusting for significant maternal factors, such as maternal age, ethnicity, occupation, parity, antenatal care, hypertensive disorders, prolonged/obstructed labour, and mode of delivery, multiple births were also more likely to be associated with moderate/extreme prematurity (<34 weeks), low birthweight (<2,500 g), IUGR, low five-minute Apgar scores (<7), neonatal sepsis, and admission to the SCBU. The risk of adverse perinatal outcomes was the highest for multiples with low birthweight (OR: 6.45, 95% CI 4.80-8.66) and IUGR (OR: 9.04, 95% CI 6.62-12.34). Although multiple births had a more than 60% increased risk of severe hyperbilirubinaemia, the association was only of borderline significance (p=0.052). In addition, multiple births were at no greater risk of sensorineural hearing loss than singletons (p=0.085). Birth asphyxia, sepsis, hyperbilirubinaemia, and admission to the SCBU were no longer significant after adjusting for prematurity, low birthweight, and IUGR. The collinearity diagnostics did not show any redundancy between gestational age and birthweight in the regression model.

The linear trend across gestational age for the neonatal outcomes showing the significant (p≤0.05) association with multiple births is presented in Table 4. The additional outcomes, such as hyperbilirubinaemia and mode of feeding, were included based on biological plausibility while the hearing status was considered within the context of the primary UNHS study. The incidence of low birthweight (<2,500 g), admission to the SCBU, and non-exclusive breastfeeding significantly declined while IUGR increased with higher gestational age. Gestational age did not have any significant linear impact among multiple births on the risk of birth asphyxia, neonatal sepsis, hyperbilirubinaemia, and status of hearing loss.

**Table 1.** Characteristics of mothers with single and multiple gestations in Lagos, Nigeria

Factor	Single pregnancies (n=4,416) No. (%)	Multiple pregnancies (n=157) No. (%)	Odds ratio (95% CI)
Sociodemographic			
Age (years)$			
<25	708 (98.2)	13 (1.8)	1.0
25-34	3,038 (96.4)	113 (3.6)	2.03 (1.13-3.62)*
>34	663 (95.7)	30 (4.3)	2.46 (1.27-4.77)**
Mean±SD	29.30±4.95	29.88±4.71
Range	13-47	19-43	-
Gestational age (weeks)			
Mean±SD	37.81±1.95	37.99±2.93	-
Range	24-50	29-50	
Ethnicity			
Yoruba	3,352 (96.8)	112 (3.2)	1.0
Non-Yoruba	1,064 (95.9)	45 (4.1)	1.27 (0.89-1.80)
Education			
None	96 (95.0)	5 (5.0)	1.0
Primary/secondary	2,620 (96.4)	99 (3.6)	0.73 (0.29-1.82)
Tertiary	1,700 (97.0)	53 (3.0)	0.60 (0.23-1.53)
Occupation			
None	869 (97.9)	19 (2.1)	1.0
Informal/part-time	2,219 (96.4)	82 (3.6)	1.69 (1.02-2.80)*
Full-time job	1,328 (96.0)	56 (4.0)	1.93 (1.14-3.27)*
Obstetric history			
Parity			
Primiparous	2,341 (99.2)	18 (0.8)	1.0
2-4	1,934 (94.3)	117 (5.7)	7.87 (4.77-12.97)***
>4	141 (86.5)	22 (13.5)	20.29 (10.64-38.70)***
Maternal HIV			
No	4,170 (96.6)	147 (3.4)	1.0
Yes	246 (96.1)	10 (3.9)	1.15 (0.60-2.22)
Antenatal care			
Yes	2,912 (97.2)	85 (2.8)	1.0
None	1,504 (95.4)	72 (4.6)	1.64 (1.19-2.26)**
Herbal drug-use			
No	3,569 (96.5)	131 (3.5)	1.0
Yes	847 (97.0)	26 (3.0)	0.84 (0.55-1.28)

$Missing data: 8 (0.2%);

*p<0.05;

**p<0.01;

***p<0.001;

CI=Confidence interval;

SD=Standard deviation

## DISCUSSION

### Principal findings

A principal finding in this predominantly Yoruba population was a high rate of multiple births of approximately 64 per 1,000 livebirths. Multiple births were more likely to be female and significantly associated with several maternal factors, such as advanced maternal age, full-time job, higher parity, lack of antenatal care, hypertensive disorders, and caesarean delivery but unlikely to be associated with prolonged/obstructed labour. Multiple births were also associated with a range of adverse perinatal outcomes that portend significant neurodevelopmental risks for the survivors, such as birth asphyxia (indexed by low 5-minute Apgar score), neonatal sepsis, and severe hyperbilirubinaemia underpinned by prematurity, low birthweight, and IUGR. The risk of adverse infant outcomes significantly reduced with increasing gestational age at delivery.

**Table 2.** Adverse obstetric events associated with multiple gestations in Lagos, Nigeria

Pregnancy-related event	Single pregnancies (n=4,416) No. (%)	Multiple pregnancies (n=157) No. (%)	Crude OR (95% CI)	Adjusted$ OR (95% CI)
Antepartum haemorrhage	47 (95.9)	2 (4.1)	1.20 (0.29-4.98)	-
Premature rupture of the membranes	47 (95.9)	2 (4.1)	1.20 (0.29-4.98)	-
Hypertensive disorders	268 (91.2)	26 (8.8)	3.07 (1.98-4.76)	3.16 (1.97-5.06)***
Prolonged/obstructed labour	413 (98.3)	7 (1.7)	0.45 (0.21-0.97)	0.46 (0.21-1.00)*
Caesarean delivery	1879 (95.5)	89 (4.5)	1.77 (1.28-2.44)	1.70 (1.21-2.38)**

$Adjusted for maternal age, ethnicity, occupation, parity, and antenatal care;

*p=0.05;

**p<0.01;

***p<0.001;

CI=Confidence interval;

OR=Odds ratio

**Table 3.** Neonatal outcomes associated with multiple gestations in Lagos, Nigeria

Outcome	Singletons (n=4416) No. (%)	Multiple births (n=302) No. (%)	Crude odds ratio (95% CI)	Adjusted OR$ (95% CI)	Adjusted OR§ (95% CI)
Prenatal					
Female sex	2,106 (92.7)	166 (7.3)	1.33 (1.07-1.66)	1.34 (1.05-1.72)*	-
Gestational age <34 weeks	136 (85.5)	23 (14.5)	2.61 (1.65-4.13)	1.91 (1.14-3.19)*	-
Gestational age<37 weeks	866 (91.6)	79 (8.4)	1.46 (1.12-1.91)	1.17 (0.87-1.57)	-
Birthweight of <1,500 g	27 (81.8)	6 (18.2)	3.31 (1.35 −8.07)	1.71 (0.66-4.45)	-
Birthweight of <2,500 g	439 (76.9)	132 (23.1)	7.17 (5.59-9.21)	6.45 (4.80-8.66)***	-
Intrauterine growth restriction	257 (71.0)	105 (29.0)	9.33 (7.11-12.25)	9.04 (6.62 minus;12.34)***	-
Perinatal					
Apgar score <7at 1 minute	3,722 (93.1)	277 (6.9)	1.50 (0.94-2.38)	-	-
Apgar score <7at 5 minutes	1,156 (91.5)	108 (8.5)	1.47 (1.15-1.88)	1.47 (1.13-1.93)**	1.15 (0.86-1.56)
Sepsis	133 (86.4)	21 (13.6)	2.41 (1.50-3.87)	2.16 (1.28-3.65)**	1.24 (0.65-2.35)
Hyperbilirubinaemia	174 (88.3)	23 (11.7)	2.01 (1.28-3.16)	1.60 (1.00-2.56)†	0.86 (0.49-1.53)
Admitted to SCBU	527 (90.2)	57 (9.8)	1.72 (1.27-2.32)	1.56 (1.12-2.17)**	0.94 (0.63-1.40)
Non-exclusive breastfeeding	763 (91.3)	73 (8.7)	1.53 (1.16-2.01)	1.34 (0.99-1.81)	1.36 (0.98-1.88)
Failed hearing tests	108 (89.3)	13 (10.7)	1.76 (0.98-3.17)	1.77 (0.93-3.37)	1.46 (0.74-2.87)

$Adjusted for maternal age, ethnicity, occupation, parity, antenatal care, hypertensive disorders, prolonged/obstructed labour, and mode of delivery;

§Adjusted for maternal age, ethnicity, occupation, parity, antenatal care, hypertensive disorders, prolonged/obstructed labor, mode of delivery, prematurity, low birthweight, and IUGR;

*p<0.05;

**p<0.01;

***p<0.001;

†p=0.052;

CI=Confidence interval;

IUGR=Intrauterine/foetal growth restriction;

OR=Odds ratio;

SCBU=Special care baby unit

**Table 4.** Trend in neonatal outcomes among multiple births by gestational age (n=302)

Outcome	<34 weeks (n=23) (%)	34-36 weeks (n=56) (%)	≥37 weeks (n=223) (%)	p value for trend
Birthweight <2.5 kg*	21 (95.5)	28 (51.9)	83 (38.2)	<0.001
Mean±SD kg	1.80±0.39	2.41±0.44	2.64±0.49	<0.001
Intrauterine growth restriction†	1 (4.5)	10 (18.5)	94 (45.6)	<0.001
Apgar score <7 at 5 minutes‡	11 (47.8)	18 (32.1)	79 (36.6)	0.604
Sepsis¶	6 (26.1)	0 (0.0)	15 (6.8)	0.074
Hyperbilirubinaemia§	1 (4.3)	3 (5.4)	19 (8.6)	0.320
Admitted to SCBU**	14 (60.9)	5 (8.9)	38 (17.2)	0.001
Non-exclusive breastfeeding††	9 (39.1)	19 (33.9)	45 (20.4)	0.008
Failed hearing tests‡‡	0 (0.0)	5 (38.5)	8 (61.5)	0.755

Missing data:

*9 (3.0%);

†20 (6.6%);

‡7 (2.3%);

¶2 (0.7%);

§2 (0.7%);

**2 (0.7%);

††2 (0.7%);

‡‡8 (2.6%);

SCBU=Special care baby unit;

SD=Standard deviation

### Comparison with other studies

The incidence of multiple gestations was significantly higher than the estimated rates of 25-50 per 1,000 livebirths for Nigeria and other developing countries (5,7-13,23). Although no data on the use of infertility treatments, such as assisted reproductive technology (ART) or ovulation stimulation, were available, multiple gestations in this population were likely to be predominantly associatedwith non-assisted ‘spontaneous’ conception as assisted/technology-driven conception is still not a widely-embraced option for many women in developing countries for economic and cultural reasons (4,7,23,24). The exceptionally-high rate of multiple births in this inner-city public hospital may be attributable to high referrals from the surrounding private hospitals and maternity homes run by traditional birth attendants (TBAs), which did not routinely admit women with multiple pregnancies because of high risk of delivery-related complications.

The increased risk of multiple gestations with advancing age and parity operating through the hypothalamic-pituitary-ovarian axis and the associated obstetric outcomes, such as hypertension, prolonged/obstructed labour, and caesarean delivery, are in agreement with reports from developing (4,11) and/or developed countries (1-3,5,7). Hypertension emerged as the most serious pregnancy-related complication consistent with vast evidence in the literature (1,3). The association between occupation and multiple gestations was rarely reported in other studies. The increased risk among mothers with full-time employment may reflect their higher economic capacity to access infertility treatments with a greater possibility of multiple gestations compared to unemployed mothers (25). However, the contribution of ART in the present study appeared uncertain (23). It is also plausible that full-time employed women in this study are more likely to be older and are, thus, at a higher risk of multiple pregnancies. It may be of interest in future studies to establish the potential link between occupational stress and increased concentration of follicle-stimulating hormone (FSH) in the mother, which has been associated with spontaneous dizygotic twining (7,8). The significantly-higher rate of the lack of antenatal care among multiparous mothers with multiple births should be of concern and deserving prompt intervention to mitigate the risk of adverse but preventable pregnancy outcomes. It was not unusual for such mothers to hold the erroneous view that they were capable of dealing with their current childbirth safely based on their past experience(s).

The findings of the present study also corroborate the extensive evidence in the literature that prematurity, low birthweight, and IUGR are the commonest immediate infant outcomes of multiple gestations worldwide (1-3,5). The increased risk of birth asphyxia/low five-minute Apgar scores, neonatal sepsis, and admission to the SCBU is also consistent with the findings of a multi-centre study by Refuerzo *et al*. on multiple gestations in the USA (26). In a case-control study in Africa, Nkyekyer also demonstrated an increased risk of neonatal admission for intensive care among twins (27). Although the association of hyperbilirubinaemia with multiple births was of a borderline significance statistically (p=0.052), the clinical significance of this condition in some special populations of twins has been reported (28,29). Thus, the finding on hyperbilirubinaemia in the present study must be interpreted with caution.

There is substantial evidence, particularly from the developing world, that the observed adverse perinatal outcomes are associated with developmental delays or long-term neurodevelopmental disabilities (30-34). For example, in one report from Kenya, neonatal jaundice and sepsis were significantly associated with neurodevelopmental deficits among a group of children who received developmental assessment between the ages of 18-32 months (34). Another report also found that a positive history of birth asphyxia, neonatal jaundice, and sepsis was significantly associated with neurological impairment among children aged 6-9 years (33). A study among a Brazilian cohort that received developmental assessment at the age of two years also found the five-minute Apgar score (<7) as a risk factor for developmental delay (32). The findings in this study would, therefore, suggest the need for close developmental surveillance of multiple births and education of parents on the importance of timely intervention for the optimal developmental outcomes in affected children.

The evidence on the mediating effects of prematurity, low birthweight, and IUGR corroborates the findings of a comparable study by Lung *et al*. (25). Conceptually, this observation suggests that the neonatal morbidities associated with multiple births can be curtailed by addressing these prenatal factors. However, several studies have demonstrated that multiple births are still independently at an increased risk of neurodevelopmental disabilities, such as cerebral palsy, learning disability, epilepsy, and overall developmental deficits even after adjusting for these factors (1-3,14,16,25). For example, there are indications that cerebral palsy in multiple births is not only attributable partly to prematurity or low birthweight but also to other factors inherent in twinning that are potential modulators of adverse neurodevelopmental outcomes in-utero, such as death of a co-twin, sex discordance, birthweight discordance, twin-twin transfusion, and ART (14). It has also been suggested that same-sex twins are at a greater risk of cerebral palsy, especially among those without a surviving second twin (16). Against this backdrop, the neurodevelopmental consequences of multiple births with or without ART, particularly in developing countries, are likely to be the product of complex interactions of diverse genetic/biological and environmental factors which are yet to be fully understood.

The evidence from the present study is significant in providing insights into the perinatal profile of multiple births, the scope of clinical support that may be required by the survivors at birth before hospital discharge, and the neurodevelopmental risk that could be anticipated from early childhood. Perhaps more so, as the complete prevention of adverse prenatal factors remains an unattainable goal worldwide and the costs associated with even apparently healthy full-term multiple births may be prohibitive in resource-poor settings (35). The significant association of multiple gestations with the female sex in this study also warrants further research considering the dearth of corroborating evidence in the literature from this region (26), although some comparable studies also found a higher proportion of female twins in their population (9,12). While no direct association was found between multiple gestations and hearing screening failure, the risk of sensorineural hearing loss secondary to ineffective or inappropriate management of the other perinatal outcomes, such as severe hyperbilirubinaemia which is not uncommon in many resource-poor settings, should not be overlooked. The weak association with congenital anomalies was possibly due to under-ascertainment of all multiple births, including stillbirths and distribution of outcomes by zygosity. For example, monozygotic twins areknown to have an elevated risk of congenital anomalies of the cardiac, renal and intestinal systems (16).

This study was conducted among an ethnic population reputed to have the highest rates of twins globally and extends the current evidence on the burden of multiple gestations in developing countries beyond the well-reported outcome on perinatal mortality. It also complements the existing evidence on the long-term outcomes of multiple births, such as cerebral palsy and learning disability worldwide, thereby underscoring the multiple pathways to neurodevelopmental compromisefrom early childhood.

### Limitations

A number of limitations are worth noting. This was a retrospective hospital-based study with an obvious selection bias, which could compromise the generalizability of the key findings, particularly on the representativeness of the reported incidence of multiple births. Lack of detailed information on the use of fertility drugs, zygosity, and chorionicity in this study limited the depth of the analysis. Gestational age was based primarily on maternal report of the LMP, which is prone to imprecision but still indispensable in clinical practice worldwide, especially in resource-constrained settings (36). The range and clinical diagnosis of morbidities examined was restricted. Considering that multiple births are also associated with an elevated risk of dying within the first year of life, the developmental trajectory of this cohort of infants still needs to be established in a longitudinal study to evaluate the neurodevelopmental burden of multiple births more accurately in any population. Notwithstanding, this study provides important baseline data to guide future work in a region of the world with the highest burden of multiple gestations and to anticipate the scope of perinatal care that may be required by multiple births more reliably in the developing world.

### Conclusions

In an ethnic population reputed to have the highest twining rates worldwide, multiple gestations were found to have an elevated risk of prematurity, low birthweight, and IUGR. These factors significantly mediated other adverse perinatal outcomes, such as low five-minute Apgar scores, neonatal sepsis, admission to the SCBU, and hyperbilirubinaemia. This study has demonstrated the need for efforts to reduce the incidence of these adverse perinatal outcomes among mothers with multiple pregnancies and minimizing the potential neurodevelopmental delays or disabilities associated with these conditions among the surviving infants in developing countries. Since the primary prevention of all prematurity, low birthweight, and IUGR remains an unattainable goal worldwide, more so in resource-poor countries, the affected mothers should be educated on the importance of timely intervention and developmental surveillance for optimal child growth and development.

## ACKNOWLEDGEMENTS

The author acknowledges the support of the entire management and staff of the Island Maternity Hospital, Lagos, Nigeria, during data retrieval for the study. The author also benefited from the constructive comments of two anonymous reviewers.
